# Edge-to-Edge mitral valve repair for preoperative bridging to heart transplantation

**DOI:** 10.1016/j.ijcha.2024.101520

**Published:** 2024-10-13

**Authors:** Moritz Messner, Simon Staggl, Maria Ungericht, Marc-Michael Zaruba, Christian Puelacher, Lukas Stastny, Jakob Hirsch, Herwig Antretter, Thomas Schuetz, Nikolaos Bonaros, Julia Dumfarth, Christoph Brenner, Axel Bauer, Gerhard Poelzl

**Affiliations:** a Department of Internal Medicine III, Cardiology and Angiology, Medical University Innsbruck, 6020 Innsbruck, Tyrol, Austria; b Department for Cardiac Surgery, Medical University Innsbruck, 6020 Innsbruck, Tyrol, Austria

**Keywords:** Mitral regurgitation, Transcatheter edge-to-edge repair, Advanced heart failure, Bridge to transplant, Bridge to candidacy

## Abstract

Transcatheter edge-to-edge repair (TEER) is a less invasive alternative to mitral valve surgery. In patients with advanced heart failure (HF), TEER can improve pulmonary hypertension (PH) and decelerate the progression of HF. TEER could be considered as a possible bridging strategy before orthotopic heart transplantation (OHT) in suitable patients. We report our experience in patients with advanced HF and severe functional mitral regurgitation (FMR) who underwent TEER prior to OHT. In this retrospective single-center study, we evaluated the periprocedural characteristics and clinical and hemodynamic outcomes of 14 patients with advanced HF on guideline-directed medical therapy and severe FMR who underwent TEER prior to OHT. In 6 patients who were not eligible for transplantation because of PH, TEER was performed as bridge-to-candidacy (BTC) strategy, in 8 unstable patients on the waiting list as bridge-to-transplant (BTT) strategy.

Severity of FMR was reduced by 2 degrees from 4 (3–4) to 2 (1.25–2.25) (p < 0.001), NYHA class from 3 (2–3) to 2 (1.75–2.13) (p = 0.003) and NT-proBNP from 4689 (2841–7932) ng/L to 2973 (1694–4812) ng/L (p = 0.008). Significant reduction in PH was observed in the BTC cohort (mean PAP from 50 (39–53) to 26 (23–33) (p = 0.027) and PCWP from to 34 (29–40) to 13.5 (11–21) mmHg (p = 0.027). 13 patients underwent successful OHT, 1 patient of the BTT cohort died of sepsis shortly after HTX listing. In conclusion patients with advanced HF and severe FMR who are considered for OHT, TEER appears suitable both as a BTC strategy in patients with PH and as a BTT strategy in unstable patients on the waiting list.

## Graphical abstract

**Figure:**
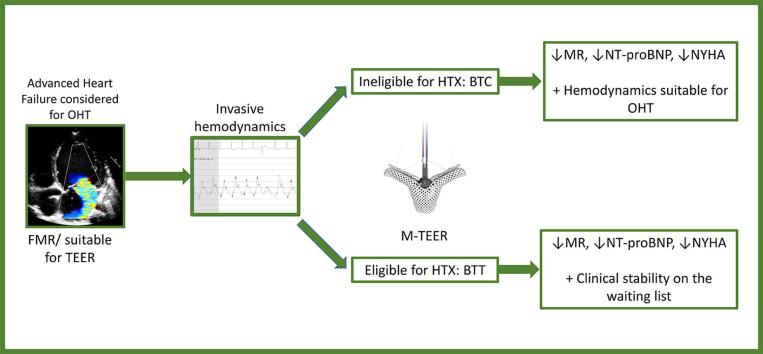

## 1 Introduction

Despite ongoing advances in heart failure (HF) therapy, the demand for heart transplants remains high [1]. Precise patient selection and meticulous pre-transplant management are pivotal for improving postoperative outcomes in this complex patient cohort [2]. A major obstacle to transplantation is pulmonary hypertension (PH), which is an exclusion criterion from transplant. [3]PH in these patients is commonly secondary to increased filling pressures from left ventricular (LV) dysfunction. Left atrial pressures can be aggravated by severe functional mitral regurgitation (FMR) secondary to ventricular and/or atrial dilatation [4]. Over time, this can cause mixed post- and precapillary PH and subsequently fixed PH [5], which is a relative or absolute contraindication to orthotopic heart transplantation (OHT). Implantation of a durable LV assist device (d-LVAD) has been shown to be effective to reduce PH and thus achieve a transplantable state in these patients. This is referred to as bridge-to-candidacy (BTC) strategy. However, this therapy is invasive, costly, and can adversely affect both the short- and long-term post-transplant survival due to the associated peri- and post-operative risks [6]. In suitable patients, the less invasive and less expensive transcatheter edge-to-edge mitral valve (TEER) may be a reasonable alternative [7].

Transplant outcomes also depend largely on the patient's mobility and clinical condition at the time of OHT. It is therefore a priority of pre-transplant management to bridge patients on the waiting list to transplantation in as stable a condition as possible, which is referred to as bridge-to-transplant (BTT) strategy. TEER has shown to be effective in largely stable patients with disproportional MR [8]. However, it is conceivable that the reduction of severe FMR using TEER can also contribute to short-term stabilization of patients on the waiting list for transplant. In this paper, we present our experience with TEER in patients with end-stage HF and severe FMR as both BTC and BTT strategy. In addition to procedural specifics, we report on the hemodynamic and clinical outcomes.

## 2 Methods

In this retrospective single-centre study 14 consecutive patients with end-stage HF and severe FMR were analysed. These patients were evaluated for, or were already on, the waiting list for OHT from 2015 to 2023. The study was approved by the Ethics Committee of the University of Innsbruck on November 2, 2017 (AN2014-0352 244/4.12) and is in compliance with the ISHLT ethics statement.

Patients were assessed before TEER and during follow‐up. Evaluations included clinical history and status, comprehensive blood tests, and transthoracic echocardiography. Right-ventricle (RV) catheterization was performed prior to TEER- procedure and during follow-up, when deemed clinically necessary.

The patient cohort consisted of 2 groups, which differed according to their respective management strategies based on RV catheter measurements. Group 1 included 6 patients who underwent TEER as a BTC due to unsuitable PH for OHT listing. These patients underwent invasive hemodynamic measurements before the procedure and during follow-up.

Group 2 consisting of 8 patients, received TEER because of clinical instability while on the waiting list for OHT as BTT strategy.

TEER was performed under the guidance of fluoroscopy and transoesophageal echocardiography according to the current state of the art. 1 to 4 clips per patient were implanted. The implantation was considered successful if the severity of the mitral regurgitation (MR) was reduced by ≥ 1 grade and/or left atrial pressure was significantly reduced.

Hemodynamic measurements were performed under local anaesthesia. Pressure measurements were calibrated to the mid-axillary line. Systolic, diastolic, and mean pulmonary artery pressure (PAP) as well as pulmonary capillary wedge pressure (PCWP) were measured via a balloon-tipped Swan-Ganz catheter. The exact placement of the balloon catheter in a right lower pulmonary artery was ensured by fluoroscopy and by the shape of the wedge pressure curve. PCWP measurements were performed in end-expiration in supine position. Measurements were averaged over 5 cardiac cycles. The Fick method was used to calculate cardiac output.

Systolic, diastolic, and mean arterial pressure were measured with an arterial catheter. The following parameters were measured by transthoracic and transoesophageal echocardiography: severity of MR, left ventricular ejection fraction (LVEF), systolic pulmonary artery pressure (sPAP), and various left ventricular dimensional indices such as end-diastolic (LVEDD) and end-systolic diameters (LVESD).

### 2.1 Statistics

Data were extracted from the clinic's database and subsequently transferred into an SPSS file for analysis. Data are presented as median along with interquartile ranges or as absolute values and percentages where suitable. Medians were compared using the Wilcoxon Signed-Rank test. We considered a p-value below 0.05 as statistically significant for differences observed between pre- and post-implantation measurements. All statistical analyses were performed using SPSS (IBM SPSS Statistics, 2022, for Windows, version 29.0, Armonk, NY). Figures were made with GraphPad Prism (version 9.4.1 for Windows, GraphPad Software, Boston, MA).

## 3 Results

14 patients (median age 57 [43–61] years; 86 % men) with end‐stage HF, severe FMR who were considered for OHT as who were already on the waiting list between 2015 and 2023 at the Medical University of Innsbruck were analysed. All patients were symptomatic with severely reduced LVEF (24 [21–27] %) despite individually optimized guideline-directed medical therapy (GDMT) and 93 % had an implanted cardiac resynchronization therapy or an implantable cardioverter defibrillator (ICD). Mean pulmonary artery pressure (PAP) and PCWP at baseline were 40 (30–51) mmHg and 29 (23–34) mmHg, respectively (Supplement Table 1a). The median follow-up time until echocardiography and assessment of clinical and laboratory parameters was 105 (70–127) days. The median time from TEER to hemodynamic re-assessment was 134 days (97–377). Clinical characteristics are depicted in Table 1, echocardiographic in Table 2 and hemodynamic data of the BTC cohort are presented in Figure 2.

**Table 1:** Clinical characteristics.

		Overall (n = 14)	BTC (N=6)	BTT (N=8)
Age at procedure, years		57 (43–61)	59 (43–62)	49 (36–54)
Male sex		12 (86 %)	5 (83 %)	7 (88 %)
BMI, kg/m^2,^		22.7 (20.7–26.7)	25.9 (21.1–31.8)	22 (20–24.3)
N° clips				
1		3 (21 %)	0 (0 %)	3 (38 %)
2		5 (36 %)	4 (67 %)	1 (13 %)
3		5 (36 %)	2 (33 %)	3 (38 %)
4		1 (7 %)	0 (0 %)	1 (13 %)
NYHA-Classification:		3 (2–3)	3.0 (2.75–3.5)	2.5 (2–3)
NYHA I		0 (0 %)	0 (0 %)	0 (0 %)
NYHA II		5 (36 %)	1 (17 %)	4 (50 %)
NYHA III		7 (50 %)	3 (50 %)	4 (50 %)
NYHA IV		2 (14 %)	2 (33 %)	0 (0 %)
Systolic blood pressure, mmHg		97 (93–106)	100 (92–107)	97 (92–105)
Diastolic blood pressure, mmHg		69 (54–72)	69 (52–73)	68 (57–71)
Heart rate, beats/min		70 (60–80)	60 (51–77)	72 (68–95)
eGFR, ml/min		50 (45–60)	56 (43–60)	49 (43–60)
NT-proBNP, ng/l		4689 (2841–7932)	3869 (2528–8007)	5796 (3174–10503)
Hypercholesterolemia		7 (50 %)	4 (67 %)	3 (38 %)
Diabetes		7 (50 %)	3 (50 %)	4 (50 %)
COPD		4 (29 %)	2 (33 %)	2 (25 %)
Previous AMI		4 (29 %)	2 (33 %)	2 (25 %)
Atrial fibrillation		5 (36 %)	2 (33 %)	3 (38 %)
Mean EuroSCORE II		3.53 (2.35–4.68)	2.99 (2.22–4.63)	3.94 (2.51–6.21)
Medication				
ACE-I/ATII		5 (36 %)	2 (33 %)	4 (50 %)
ARNI		9 (64 %)	4(78 %)	4 (50 %)
MRA		13 (93 %)	6 (100 %)	7 (88 %)
Beta-blocker		12 (86 %)	5 (83 %)	7 (88 %)
Diuretics		14 (100 %)	6 (100 %)	8 (100 %)
Ivabradine		6 (43 %)	3 (50 %)	3 (38 %)
Values are presented as median and interquartile range. p-values indicate differences using Wilcoxon Signed-Rank test. NYHA: New York Heart Association class, ACE-I: Angiotensin-Converting Enzyme Inhibitor, AMI: Acute Myocardial Infarction, ARB: Angiotensin Receptor Blocker, ARNI: Angiotensin Receptor-Neprilysin Inhibitor, BMI: Body-Mass-Index, CRT: Cardiac Resynchronization Therapy, COPD: Chronic Obstructive Pulmonary Disease, eGFR: estimated Glomerular Filtration Rate, ICD: Implantable Cardioverter Defibrillator, NT-proBNP: N-terminal pro-brain natriuretic peptide.

**Table 2:** Echocardiographic characteristics

	All Baseline	FUP	P-Value	BTC Baseline	FUP	P-Value	BTT Baseline	FUP	P-Value
Median time from TEER, days	105 (70.5–127)			90 (67–102.5)			124.5 (79.5–133.75)		
MR Grade	4 (3–4)	2 (1–2.25)	**<0.001**	3.5 (3–4)	2 (1–2.25)	**0.023**	4 (3–4)	2 (1.25–2.75)	**0.010**
Grade I	0 (0 %)	2 (14 %)		0 (%)	2 (33 %)		0 (0 %)	2 (25 %)	
Grade II	0 (0 %)	10 (71 %)		0 (%)	3 (50 %)		0 (0 %)	4 (50 %)	
Grade III	6 (43 %)	3 (%)		3 (50 %)	1 (17 %)		3 (38 %)	2 (25 %)	
Grade IV	8 (57 %)			3 (50 %)	0 (0 %)		5 (63 %)	0 (0 %)	
LVEDD, mm	74 (71–86)	73 (70–80)	0.778	78 (67–92)	73 (68–82)	0.249	73 (69–83)	73 (71–84)	0.263
LVESD, mm	67 (60–76)	64 (55–74)	0.638	74 (53–83)	62 (52–74)	0.248	64 (60–69)	64 (57–76)	0.263
sPAP, mmHg (n = 12)	60 (50–61)	40 (32–54)	**0.004**	61 (59–66)	51 (31–58)	0.078	50 (45–60)	38 (34–43)	0.027
LVEF, %	24 (21–27)	22 (15–27)	0.510	25 (23–29)	15 (13–27)	**0.046**	22 (20–26)	24 (20–28)	0.161
MVG, mmHG	1.5 (1–2.25)	3 (2–4)	**0.007**	2 (1–2.25)	2.5 (1.75–2.5)	0.180	1 (1–2.75)	3 (2.25–4.5)	**0.016**
TAPSE, mm	20 (17–22)	19 (16–22)	0.657	22.5 (19–27)	20 (18–23)	0.112	18 (15.5–20)	17.5 (14–21.3)	0.526

Values are presented as median and interquartile range. p-values indicate differences using Wilcoxon Signed-Rank test.

FUP: follow-up, LVEF: Left Ventricular Ejection Fraction, LVEDD: Left Ventricular End-Diastolic Diameter, LVESD: Left Ventricular End-Systolic Diameter, MVG: Mean Mitral Valve Gradient, sPAP: Systolic Pulmonary Artery Pressure (ALL: n = 12, BTT: n = 6), TAPSE: Tricuspid Annular Plane Systolic Excursion.

**Fig. 1:**
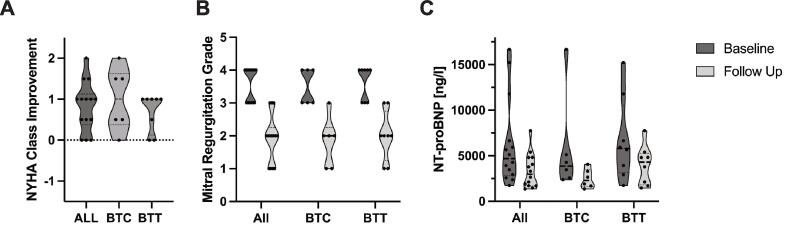
Follow-up (FUP) echocardiography (Fig. 1A) showed a significant reduction in MR from 4 (3–4) to 2 (1–2.23) (n = 14; p < 0.001). The median MR grade improved from 3.5 (3–4) to 2.0 (1–2.35) (n = 6; p = 0.023) in BTC patients and from 4 (3–4) to 2 (1.25–2.75) (n = 8; p = 0.01) in BTT patients.The median NYHA functional class (Fig. 1B) improved from class 3 (2–3) to class 2 (1.75–2.13) in all patients (n = 14; p = 0.001). NYHA class improved from 3 (2.75–3.5) to 2 (1.75–2.5) (n = 6; p = 0.041) in the BTC group and from 2.5 (2–3) to 2 (1.25–2) (n = 8; p = 0.02) in BTT patients. Overall NT-proBNP levels (Fig. 1C) decreased from 4689 (2841–7931) to 2973 ng/l (1694–4812) (n = 14; p = 0.008) after TEER. While the reduction in the BTC group from 3869 (2528–8007) to 2281 ng/l (1603–3483) was statistically significant, the numerical decrease in BTT patients from 5794 (3174–10503) to 4301 ng/l (1802–5263) was not statistically significant. Medians were compared using the Wilcoxon Signed-Rank test.

One or more clips per patient (1–2 clips: 57 %, 3 clips: 36 %, 4 clips: 7 %) were implanted. Post-procedural echocardiography revealed significant improvement of MR (4 [3], [4] to 2 [1–2.25], p < 0.001). All patients had a reduction in MR of at least one grade (Fig. 1A). At follow-up, median NYHA functional improved from class 3 (2–3) to class 2 (1.75–2.13) (p = 0.003) (Fig. 1B) and the median NT-proBNP level decreased from 4689 (2841–7932) to 2973 (1694–4812) ng/l (p = 0.008) (Fig. 1C). All but one patient, who died of sepsis 2 months after TEER, were successfully transplanted.

**Fig. 2:**
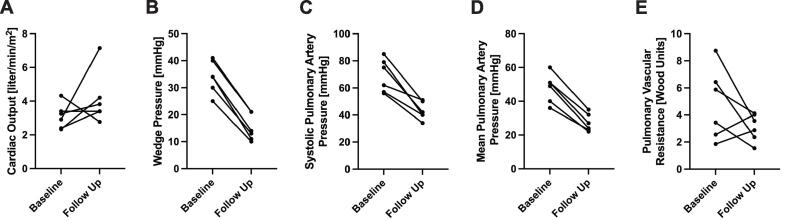
Hemodynamic measurements in BTC patients. Paired data plots showing change patterns from baseline to follow up for CO, PCWP, sPAP, mPAP and PVR (n = 6). P-values indicate differences using Wilcoxon Signed-Rank test. CO: Cardiac Output, mPAP: mean Pulmonary Artery Pressure, PCWP: Pulmonary Capillary Wedge Pressure, sPAP: systolic Pulmonary Artery Pressure, PVR: Pulmonary Vascular Resistance, sPAP: systolic Pulmonary Artery Pressure.

Invasive hemodynamic analysis was repeated in 11 patients within 134 days (97–377) after TEER. The median PCWP decreased significantly from 29 (23–34) to 21 (13–27) mmHg (p = 0.047) (Supplement Table 1) whereas the numeric decrease of mean PAP 40 (30–51) to 32 (24–35) mmHg was not statistic significant (p = 0.062). Cardiac output (CO) numerically increased from 3.1 (2.4–4.3) to 3.9 l/min (3.4–4.2) (p = 0.114).

For detailed analysis, patients were divided into a BTC and a BTT group.

### 3.1 Bridge*-to-candidacy*

TEER was performed in 6 patients (age 59 [43–62] years, 83 % men) with severe FMR and severe mixed PH unsuitable for OHT Patients presented mostly with NYHA functional class III/IV symptoms (83 %) and elevated levels of NT-proBNP (3869 [2528–8007] ng/l) (Table 1).

Two clips were implanted in 4 patients (67 %) and 3 clips in 2 patients (33 %). TEER resulted in a significant reduction in MR, which was decreased from grade 3.5 (3–4) to 2.0 (1–2.35) (p = 0.023) (Fig. 1A). There was an improvement of two NYHA functional classes in half of the patient (Fig. 1B), which was mirrored by a significant reduction in NT-proBNP to 2281 (1603–3483) ng/l (p = 0.046) (Fig. 1C).

Follow-up right heartcatheterization showed significant improvements in mean PAP from 50 (39–53) to 26 mmHg (23–33) and PCWP from 34 (29–40) to 13.5 (11–21) mmHg (Supplement Table 1b, Fig. 2). The median time from TEER to RV-catheterization was 97.5 days (27–118). While there was a tendency towards improved cardiac output (CO), this trend did not reach statistical significance (median changing from 3.08 (2.83–3.63) to 3.88 (3.24–4.94) l/min (p = 0.225). Similarly, there was no significant improvement in pulmonary vascular resistance (PVR), with values shifting from 4.66 (2.37–7.02) to 3.22 (2.15–4.05) wood unit (WU) (p = 0.116).

At follow-up, all patients were deemed suitable for heart transplantation.

### 3.2 *Bridge-*to*-transplant (n = 8)*

Eight patients (median age 49 years [36–54], 88 % men) underwent TEER in a bridge-to-transplant strategy. Patients presented with NYHA functional class II (50 %) or III symptoms (50 %), and the median NT-proBNP was 5796 (3174–10503) ng/l. All patients underwent TEER, with 1–2 clips implanted in 4 patients (50 %) 3 clips in 3 patients (38 %) and 4 clips in 1 patient (13 %) (Table 1). The median follow-up time for assessment of clinical and laboratory parameters and for echocardiography was 125 days (80–134). The median time from TEER to hemodynamic re-assessment was 377 days (253–782). The median MR severity improved from 4 (3–4) to 2 (1.25–2.75) (p = 0.01) (Fig. 1A). NYHA functional class significantly improved from 2.5 (2–3) to 2 (1.25–2) (p = 0.02), with 3 patients (38 %) maintaining the same status and 5 patients (63 %) showing an improvement of one class. NT-proBNP level was reduced from numerically from 5796 (3174–10503) to 4301 (1802–5263) (p = 0.069) (Fig. 1C).

RV- catheterization was performed in 5 out of 8 patients following clip implantation, showing no significant changes in pulmonary pressures (mean PAP: p = 0.343, PCWP: p = 0.546, or cardiac output (p = 0.686) (Supplement Table 1c).

## 4 Discussion

This retrospective analysis of patients with percutaneous mitral valve repair with advanced HF and severe FMR considered for OHT suggests that this therapy may be feasible. TEER was safely and successfully performed in patients with severe PH who were not eligible for OHT and in unstable patients on the waiting list for OHT.

FMR substantially contributes to high mortality in patients with advanced HF [9] and aggravates PH secondary to left heart disease [5].

For potential OHT candidates with PH, reducing PVR is crucial and a prerequisite for transplant eligibility. Implantation of a centrifugal, durable LVAD can effectively reverse PH [10]. However, the implantation of an LVAD is a complex and costly procedure which potentially increases perioperative and postoperative complications in OHT [6].

In our BTC group, TEER had a sustained hemodynamic effect. PH was lowered in all patients to become eligible for OHT and could ultimately be successfully transplanted.

In contrast to BTC, the focus for unstable patients who are on the waiting list for OHT is on ensuring clinical stability until transplantation. This was achieved in all patients in our BTT group with TEER, due to reduction of MR and not least by further dose optimization of GDMT in some patients, which became possible because of a slight increase in blood pressure. The effect was obvious by an improvement in the NYHA class and a decrease in NT-proBNP levels. A comparable effect on NT-proBNP was found by Tanaka et al. In this study the decrease in NT-proBNP was associated with improvements in clinical outcomes within two years after TEER. The authors suggested that NT-proBNP dynamics may be well suited for the assessment residual risks in patients undergoing TEER and to assist in post-procedural management [11].

Except for one patient who died of sepsis during the waiting period, all patients underwent successful OHT.

Over the past decade, TEER has proven to be a safe and effective therapy in patients with stable HF and severe MR [8]. In agreement with this Romano et al showed that TEER was associated with improved LA function, an improvement in cardiac performance and a reduction in arrhythmic burden [12]. However, detailed analyses of previous landmark trials showed no long-term success in patients with advanced remodelling processes in the left ventricle[13]. For this reason, patients with so-called disproportionately severe MR, i.e. severe MR with a still moderately dilated ventricle (LVEDV≤200 mL), are primarily selected for this procedure [14]. This concept however was called into question in a recent paper by Doldi et al for patients with atrial FMR[15]. Likewise, lower myocardial energetic efficiency in the context of chronic preload increase might impact the LV-response to MR correction[16].

However, the long-term effect of TEER plays a lesser role in patients with FMR being considered for OHT. In these patients, eligibility for OHT (BTC) or clinical stability until OHT (BTT) is the primary goal. In fact, all patients in our cohort already had a markedly dilated left ventricle (LVEDD 74 [71–86], LVESD 67 [60–76] mm) before TEER. Accordingly, most patients required implantation of ≥ 2 clips. No significant reduction in FMR could be achieved in two patients. In these patients the implantation of additional clips was limited by mitral valve gradient (MVG) elevations. The slight increase in mean MVG in our patient along with the natural progression of the disease, may have contributed to the observed numerical rise in PCWP in the BTT cohort..

The septal access in TEER often creates an artificial atrial septal defect (ASD) with a left-to-right shunt. This can cause beneficial hemodynamic effects in patients with HF and reduced ejection fraction [17], [18]. This phenomenon may have had an additional positive impact on the course of the disease in our patients.

The optimal antithrombotic therapy of patient undergoing TEER is still under discussion. Our patients on the waiting list for OHT were treated with vitamin K antagonists. This is in line with data from Waechter et al indicating a possible benefit of OAC after TEER [19].Standard 3D imaging was utilized during the procedure in our patients. Future advancements in imaging technology may enable more accurate assessment of the mitral valve, leaflet grasping, and residual MR jets following mitral valve repair, ultimately leading to better outcomes. Promising innovations include 3D TEE transillumination rendering (3DTr) and the integration of glass effects to adjust tissue transparency, thereby enhancing depth perception (3DG1) [20].

Our data suggest that TEER is safe and effective for appropriate patients and may be considered for OHT in a BTC or BTT situation. However, larger, prospective studies are needed to confirm this hypothesis and to identify potential responders. Testing the reversibility of PVR to afterload reduction could be useful.

## 5 Limitations

The small number of patients and the retrospective nature of the observational cohort study are clear limitations. The duration from TEER to invasive hemodynamic monitoring was variable and hemodynamic monitoring is not available for all patients in the BTT group.

The potential effect of the iatrogenic ASD, which is created *peri*-procedurally, could introduce confounding bias that cannot be excluded.

Since recruitment of our study cohort began before SGLT-2 inhibitors were added to GDMT, only five patients were on SGLT-2 inhibitor therapy prior to OHT. This may be a limitation, as it affects the generalizability and interpretation of the study findings.

A further limitation to be mentioned is the fact that performance data such as the 6-minute walk test or cardiopulmonary exercise testing were not routinely collected.

## 6 Conclusion

In patients with advanced HF and severe FMR who are considered for OHT, TEER appears suitable and effective both as a BTC strategy in patients with PH and as a BTT strategy in unstable patients on the waiting list.

## CRediT authorship contribution statement

**Moritz Messner:** Writing – original draft, Visualization, Software, Resources, Investigation, Formal analysis, Data curation, Conceptualization. **Simon Staggl:** Writing – review & editing, Visualization, Software, Investigation, Formal analysis, Data curation, Conceptualization. **Maria Ungericht:** Writing – review & editing, Methodology, Investigation, Formal analysis. **Marc-Michael Zaruba:** Writing – review & editing, Software, Resources, Investigation, Funding acquisition. **Christian Puelacher:** Writing – review & editing, Software, Methodology, Investigation, Formal analysis. **Lukas Stastny :** Writing – review & editing, Visualization, Investigation, Formal analysis. **Jakob Hirsch:** Writing – review & editing, Software, Investigation, Formal analysis, Data curation. **Antretter Herwig:** Resources, Methodology, Investigation, Conceptualization. **Thomas: Schuetz** Writing – review & editing, Visualization, Resources, Investigation, Data curation. **Nikolaos Bonaros:** Writing – review & editing, Resources, Methodology, Investigation, Conceptualization. **Julia Dumfarth :** Supervision, Methodology, Investigation, Conceptualization. **Christoph Brenner:** Writing – review & editing, Validation, Resources, Methodology, Investigation. **Axel Bauer:** Writing – review & editing, Supervision, Resources, Funding acquisition, Formal analysis, Conceptualization. **Gerhard Poelzl:** Writing – review & editing, Writing – original draft, Supervision, Resources, Methodology, Investigation, Formal analysis, Data curation, Conceptualization.

## Declaration of competing interest

The authors declare that they have no known competing financial interests or personal relationships that could have appeared to influence the work reported in this paper.
