# Supplementary material for: Edge-to-Edge mitral valve repair for preoperative bridging to heart transplantation
Source: Int J Cardiol Heart Vasc. 2024 Oct 13;55:101520. doi: 10.1016/j.ijcha.2024.101520 (PMC11795678; doi:10.1016/j.ijcha.2024.101520)
Supplement: Supplementary Data 1 [file mmc1.docx]

Supplement Table 1a: Hemodynamic measurements in all patients (n=11)

| Right ventricular catheterization characteristics | ALL  Baseline | follow-up | p-value |
| --- | --- | --- | --- |
| Median time from TEER, days | 134 days (97-377) | |  |
| Cardiac Output, l/min | 3.1 (2.4-4.3) | 3.9 (3.4-4.2) | .114 |
| Cardiac Index, l/min/m² | 1.6 (1.4-2.3) | 2.0 (1.7-2.3) | .477 |
| PCWP, mmHg | 29 (23-34) | 21 (13-27) | **.047** |
| Systolic PAP, mmHg | 56 (48-75) | 50 (40-54) | .109 |
| Diastolic PAP, mmHg | 26 (23-42) | 21 (14-26) | **.037** |
| Mean PAP, mmHg | 40 (30-51) | 32 (24-35) | .062 |
| Mean RAP, mmHg | 11 (7-14) | 9 (4-12) | .533 |
| PVR, Wood Units | 2.6 (1.9-5.9) | 2.4 (1.8-3.7) | .182 |

Supplement Table 1b: Hemodynamic measurements in bridge-to-candidacy patients (n=6)

| Right ventricular catheterization characteristics | Bridge-to-candidacy  Baseline | follow-up | p-value |
| --- | --- | --- | --- |
| Median time from TEER, days | 98(27-118). | |  |
| Cardiac Output, l/min | 3.1 (2.4-3.6) | 3.9 (3.2-4.9) | .225 |
| Cardiac Index, l/min/m² | 1.5 (1.2-1.85) | 1.9 (1.6-2.5) | .249 |
| PCWP, mmHg | 34 (29-40) | 13.5 (10-21) | **.027** |
| Systolic PAP, mmHg | 69 (57-81) | 41 (39-50) | **.028** |
| Diastolic PAP, mmHg | 38 (25-46) | 20 (13-23) | **.028** |
| Mean PAP, mmHg | 50 (39-53) | 26 (23-33) | **.027** |
| Mean RAP, mmHg | 12.5 (8.5-14) | 4.5 (3.8-11) | **.046** |
| PVR, Wood Units | 4.7 (2.4-7) | 3.22 (2.2-4.1) | .116 |

Supplement Table 1c: Hemodynamic measurements in bridge-to-transplant patients (n=5)

| Right ventricular catheterization characteristics | Bridge-to-transplant  baseline | Follow-up | p-value |
| --- | --- | --- | --- |
| Mean time from TEER, days | 377 (252-782) | |  |
| Cardiac Output, l/min | 3.1 (2.2-4.4) | 4 (3-5.3) | .686 |
| Cardiac Index, l/min/m² | 1.9 (1.48-2.61) | 2.06 (1.53-2.63) | .686 |
| PCWP, mmHg | 23 (17-29) | 27 (21-31) | .465 |
| Systolic PAP, mmHg | 48 (34.5-52) | 54 (44.5-57.5) | .343 |
| Diastolic PAP, mmHg | 25 (15.5-28) | 25 (16.5-31) | .686 |
| Mean PAP, mmHg | 30 (23.5-38) | 34 (29-40) | .343 |
| Mean RAP, mmHg | 8 (4-12) | 10 (8-20) | .345 |
| PVR, Wood Units | 2.2 (1.7-3.2) | 2 (1.6-3) | .893 |

| Values are presented as median and interquartile range. p-values indicate differences using Wilcoxon Signed-Rank test.  PAP: Pulmonary Artery Pressure, PCWP: Pulmonary Capillary Wedge Pressure, PVR: Pulmonary Vascular Resistance, RAP: Right Atrial Pressure. |
| --- |
